# HIV-1 Subtype F1 Epidemiological Networks among Italian Heterosexual Males Are Associated with Introduction Events from South America

**DOI:** 10.1371/journal.pone.0042223

**Published:** 2012-08-02

**Authors:** Alessia Lai, Francesco R. Simonetti, Gianguglielmo Zehender, Andrea De Luca, Valeria Micheli, Paola Meraviglia, Paola Corsi, Patrizia Bagnarelli, Paolo Almi, Alessia Zoncada, Stefania Paolucci, Angela Gonnelli, Grazia Colao, Danilo Tacconi, Marco Franzetti, Massimo Ciccozzi, Maurizio Zazzi, Claudia Balotta

**Affiliations:** 1 Department of Biomedical and Clinical Science, Infectious Diseases and Immunopathology Section, ‘L. Sacco’ Hospital, University of Milan, Milan, Italy; 2 Second Infectious Diseases Unit, Siena University, Siena, Italy; 3 Laboratory of Microbiology, ‘L. Sacco’ Hospital, Milan, Italy; 4 Second Division of Infectious Diseases, ‘L. Sacco’ Hospital, Milan, Italy; 5 Unità Operativa Complessa of Infectious Diseases Careggi, Florence, Italy; 6 Department of Biomedical Science, Section of Microbiology, Laboratory of Virology, Università Politecnica delle Marche, Ancona, Italy; 7 Unità Operativa Complessa of Infectious Diseases and Hepatology, Azienda Ospedaliera Universitaria Senese, Siena, Italy; 8 Infectious Diseases Section, Cremona, Italy; 9 Struttura Semplice di Virologia Molecolare, Fondazione IRCCS Policlinico San Matteo, Pavia, Italy; 10 Section of Virology Careggi, Florence, Italy; 11 Infectious Diseases Section, Arezzo, Italy; 12 Department of Infectious, Epidemiology Unit, Parasite and Immune-Mediated Diseases, Italian Institute of Health, Rome, Italy; 13 Department of Biotechnology, Section of Microbiology, University of Siena, Siena, Italy; Public Health Agency of Barcelona, Spain

## Abstract

About 40% of the Italian HIV-1 epidemic due to non-B variants is sustained by F1 clade, which circulates at high prevalence in South America and Eastern Europe. Aim of this study was to define clade F1 origin, population dynamics and epidemiological networks through phylogenetic approaches. We analyzed *pol* sequences of 343 patients carrying F1 subtype stored in the ARCA database from 1998 to 2009. Citizenship of patients was as follows: 72.6% Italians, 9.3% South Americans and 7.3% Rumanians. Heterosexuals, Homo-bisexuals, Intravenous Drug Users accounted for 58.1%, 24.0% and 8.8% of patients, respectively. Phylogenetic analysis indicated that 70% of sequences clustered in 27 transmission networks. Two distinct groups were identified; the first clade, encompassing 56 sequences, included all Rumanian patients. The second group involved the remaining clusters and included 10 South American Homo-bisexuals in 9 distinct clusters. Heterosexual modality of infection was significantly associated with the probability to be detected in transmission networks. Heterosexuals were prevalent either among Italians (67.2%) or Rumanians (50%); by contrast, Homo-bisexuals accounted for 71.4% of South Americans. Among patients with resistant strains the proportion of clustering sequences was 57.1%, involving 14 clusters (51.8%). Resistance in clusters tended to be higher in South Americans (28.6%) compared to Italian (17.7%) and Rumanian patients (14.3%). A striking proportion of epidemiological networks could be identified in heterosexuals carrying F1 subtype residing in Italy. Italian Heterosexual males predominated within epidemiological clusters while foreign patients were mainly Heterosexual Rumanians, both males and females, and South American Homo-bisexuals. Tree topology suggested that F1 variant from South America gave rise to the Italian F1 epidemic through multiple introduction events. The contact tracing also revealed an unexpected burden of resistance in epidemiological clusters underlying the need of public interventions to limit the spread of non-B subtypes and transmitted drug resistance.

## Introduction

HIV-1 is characterized by a high evolutionary rate that led to the establishment of different viral lineages. Group M is the most widespread group representing the main source of the HIV/AIDS pandemic. Based upon latest classification, it is further divided into nine subtypes (A, B, C, D, F, G, H, J and K), six sub-subtypes (A1, A2, A3, A4, F1 and F2), an increasing number of Circulating Recombinant Forms (CRFs) (of whom 52 are known at present) and an undetermined number of Unique Recombinant Forms (URFs) [1].

As a consequence of migratory waves from low-middle income areas, non-B variants entered and circulates in almost all previously B-restricted European countries [2]–[6]. The spread of non-B clades in Italy occurred in conjunction with relevant epidemiological changes such as the increase of sexual transmission [7]. Indeed, the prevalence of non-B subtypes grew from 2.6% in 1985–1992 to 18.9% in 1993–2008. Among these non-B isolates, subtype F1 is the most frequent variant (44.3%) among European subjects followed at 50 Italian clinical Centers [8].

HIV-1 F1 subtype was firstly isolated in the Democratic Republic of Congo, where it accounts for a small percentage (<5%) of diagnosed cases at present [9]. This subtype circulates at low frequency worldwide (<1%) and has spread to South America and Europe, both as a ‘pure’ strain and as part of several B/F recombinant forms [10]. In South America, F1 subtype and BF recombinant forms account for more than 10% of HIV-1 epidemic and are associated with intravenous drug use and heterosexual route of infection [11].

In Europe, subtype F1 has a massive prevalence in Rumania (>70%), where it spread among adults few years before its striking emergence among institutionalized children around 1989 [12].

The origin of HIV-1 non-B infections in Italy has been not thoroughly investigated by specific population studies [13]–[15]. Information on epidemiological networks has been obtained in small areas or in B-subtype restricted population [16], [17].

Since HIV-1 gene sequencing for drug-resistance monitoring has been widely adopted, new phylogenetic methods have been developed which allow to infer evolutionary dynamic from sequence data [18], [19]. Molecular epidemiology may trace the origin of viral infections, reveal outbreaks within population subgroups and provide a means for monitoring the spread within and among groups with different mode of infection. Moreover, phylogenetic analysis can investigate evolutionary patterns of pathogens within a specific population, gaining useful insights on their epidemics [20].

The burden of immigration in Italy, reported by the National Institute for Statistics (ISTAT) (www.istast.it), indicates that Rumanians with about 1 million of persons, account for the main foreign community, while no information are available for specific countries of South America that contribute to immigration with about 350,000 presences. The aim of this study was to reconstruct the epidemiological features of F1 subtype in Italy by analyzing data from a nation-wide collaboration. Furthermore, we identified the epidemiological networks and established the relationships among the main demographic variables such as citizenship, route of infection and gender.

## Patients and Methods

### Study Population

We analyzed 343 individuals carrying HIV-1 F1 variant, who had been referred to 50 clinical Centers in 13 Italian regions in the period 1998–2009. Regarding the geographical proportion of individuals, 160, 161 and 22 subjects were followed at Clinical Centers of North, Central and South of Italy, respectively.

Patients received a genotypic resistance assays at diagnosis or prior the start of therapy or at treatment failure. Tests were performed at local Services of participating clinical Centers. For each patient included in the analysis, the earliest available HIV-1 genotype was evaluated. All patients are included in the Antiretroviral Resistance Cohort Analysis (www.hivarca.net) and in a dedicated database in Milan. They provided an informed written consent to have their anonymized data stored on a central server and used for academic studies. Informed consent statements are available at each clinical Centre. Demographic data (gender, risk category, country of origin, date of diagnosis and age) were collected by physicians from patient interviews and recorded in the databases together with virological, immunological and treatment information. The authors were exempted from approval by Ethic Committee of ‘L. Sacco’ Hospital because no human experimentation and no investigation of host genetics were conducted. The study was conducted in accordance with the 1964 Declaration of Helsinki and the ethical standards of the Italian Ministry of Health.

### Phylogenetic Analysis

The Bayesian phylogenetic tree was reconstructed by means of MrBayes using a general-time reversible (GTR) model of nucleotide substitution, a proportion of invariant sites, and gamma distributed rates among sites [21]. A Markov Chain Monte Carlo (MCMC) search was made for 10×10^6^ generations using tree sampling every 100th generation and a burn-in fraction of 50%. Statistical support for specific clades was obtained by calculating the posterior probability of each monophyletic clade, and a posterior consensus tree was generated after a 50% burn-in. Clades with a posterior probability of 1 were considered epidemiological clusters. The posterior consensus tree obtained by means of MrBayes was used as the starting tree.

The dated trees, evolutionary rates and population growth were co-estimated by using a Bayesian MCMC approach (Beast version 1.5.4 http://beast.bio.ed.ac.uk) implementing the GTR+Invariant+Gamma model [22]. We compared different parametric demographic models (a constant population size, exponential and logistic growth) and a Bayesian skyline plot (BSP) under strict and relaxed clock conditions. The best model was selected by means of the Bayes factor (BF), using marginal likelihoods and implemented in Beast [23]. In accordance with Kass and Raftery, the strength of the evidence against Hypothesis 0 (H0) was evaluated as follows: 2lnBF <2 no evidence; 2–6 weak evidence; 6–10 strong evidence; >10 very strong evidence. A negative value indicates evidence in favor of H0 [22]. Only values ≥6 were considered significant. Chains were conducted for at least 300×10^6^ generations, and sampled every 30,000 steps. Convergence was assessed on the basis of the effective sampling size (ESS) after a 10% burn-in using Tracer software version 1.5 (http://tree.bio.ed.ac.uk/software/tracer/). Only parameter estimates with ESS’s >200 were accepted. Uncertainty in the estimates was indicated by 95% highest posterior density (95% HPD) intervals. Trees were summarized in a target tree by Tree Annotator program included in the Beast package choosing the tree with the maximum product of posterior probabilities (maximum clade credibility) after a 50% burn-in.

### Statistical Methods

Categorical variables were summarized as frequencies and comparisons between groups were performed using the χ^2^ or the Fisher exact test. For all the analyses an α error of 5% was considered. Analyses were performed using the SPSS software package (v.16.0, SPSS Inc. Chicago, IL, USA).

## Results

### Characteristics of Population

We studied 343 HIV-1 F1 infected patients with sequence data obtained from 1998 to 2009. According to country of origin, 72.6% (n = 249), 9.3% (n = 32), 7.3% (n = 25) and 10.8% (n = 37) of patients were Italians, South Americans, Rumanians and other/unknown, respectively. Patients from South America had Brazil (n = 25), Paraguay (n = 3), Argentina (n = 2) and Columbia (n = 2) as country of origin.

Among patients with known modality of infection (n = 217), heterosexuals (HETs), homo-bisexuals (HO-BISEXs), intravenous drug users (IDUs) and others accounted for 58.1% (n = 126), 24.0% (n = 52), 8.8% (n = 19) and 9.2% (n = 20) of patients, respectively. The heterosexual contacts were significantly more frequent among Italians with known risk factor (103/153, 67.3%) and Rumanians (7/14, 50%), than among South Americans, who were mainly HO-BISEXs (16/18, 88.9%) (*p*<0.001).

Overall, three out of four subjects were males (n = 257, 75.8%); the male to female ratio was about 4∶1, 7∶1 and 1∶1.5 among Italians, South Americans and Rumanians, respectively. Males were predominant among Italians and South Americans but not among Rumanians (87.5% and 79.8% *vs.* 40%, respectively*; p*<0.001).

The median age was 38 years (min-max 10–73 years); HIV-1 viral load levels and CD4 cells ranged from 2 to 6 log copies/ml and from 3 to 1.990 cells/µL (medians 4.5 log copies/ml and 331 cells/µL), respectively.

### Phylogenetic Analysis

The Bayesian tree of HIV-1 F1 sequences is shown in Figure S1. We identified 27 significant epidemiological networks with posterior probability of 1. Clusters involved 70.0% of sequences (n = 240). A variable number of isolates was included in distinct clades (ranging from 3 to 56); 6 of them included more than 10 sequences.

The epidemiological characteristics of all the patients and of the individuals included in the epidemiological networks are reported in Table 1.

**Table 1:** Study population and epidemiological network: risk factor and gender according to the most frequent citizenship of patients (n = 306).

		ITALIANS		SOUTH AMERICANS		RUMANIANS	
		81.4% (n = 249)		10.4% (n = 32)		8.2% (n = 25)	
		Total	Clustering	Total	Clustering	Total	Clustering
		% (n)	% (n)	% (n)	% (n)	% (n)	% (n)
**RISK FACTOR** 1	**HETs** 2	67.3 (103)	79.6 (82)	11.1 (2)	100.0 (2)	50.0 (7)	100.0 (7)
	**Homo-bisexuals** 3	20.3 (31)	77.4 (24)	88.9 (16)	31.3 (5)	–	–
**GENDER** 4	**Males**	79.5 (198)	73.2 (145)	87.5 (28)	28.6 (8)	40.0 (10)	100.0 (10)
	**Females**	20.1 (50)	76.0 (38)	12.5 (4)	50.0 (2)	60.0 (15)	100.0 (15)

1 Risk factor was known for 153 Italians, 18 South Americans and 14 Rumanians.

2 HETs: heterosexuals.

3 HO-BISEXs: homo-bisexuals.

4 Gender was known for 248 Italians, 32 South Americans and 25 Rumanians.

The heterosexual modality of infection was significantly associated with the probability to be detected in networks (78.6% HETs vs. 63.5% HO-BISEXs, *p* = 0.04). No association was detected between gender and clustering probability.

Within the epidemiological networks, heterosexual risk factor was predominant among Italian (82/122, 67.2%) and Rumanian patients (7/14, 50%), whereas homo-bisexual contacts were prevalent among South Americans and accounted for 5/7 (71.4%) of these patients. Therefore, a different distribution of modality of infection was present in clusters according to citizenship (*p*<0.001). As a consequence, a significant difference could be detected for gender distribution among distinct citizenships in networks; while males were prevalent among Italians (79.2%) and South Americans (80%), females accounted for 60% of Rumanians (*p*<0.001).

### Evolutionary Rate Estimation

In order to obtain a time scaled phylogeny, we estimated the evolutionary rate based on known sampling date of our sequences. The comparison of the different models by BF test, shown in Table S1, selected a Bayesian skyline plot (BSP) model for the population dynamics under a relaxed (lognormal) clock (2lnBF relaxed  = 352.4). Under these conditions, we estimated a mean evolutionary rate of 1.8×10^−3^ substitution/site/year (credibility interval between 2.65×10^−3^ and 8.18×10^−4^ substitution/site/year) for the HIV-1 F1 *pol* sequences. On the basis of this value we estimated the Time of Most Recent Common Ancestor (TMRCA) of the root and every internal nodes of the tree.

### Time Scaled HIV-1 F Phylogeny

**Figure 1:**
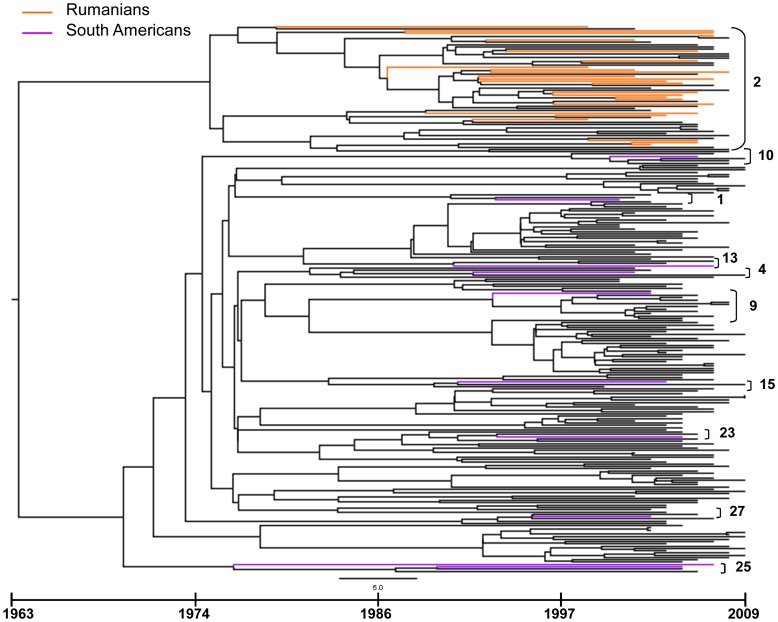
Dated tree showing clustering sequences. Cluster numbers are shown on the right. Time-line scale is displayed underneath the tree. Different nationalities of patient isolates is indicated by different colors.

**Figure 2:**
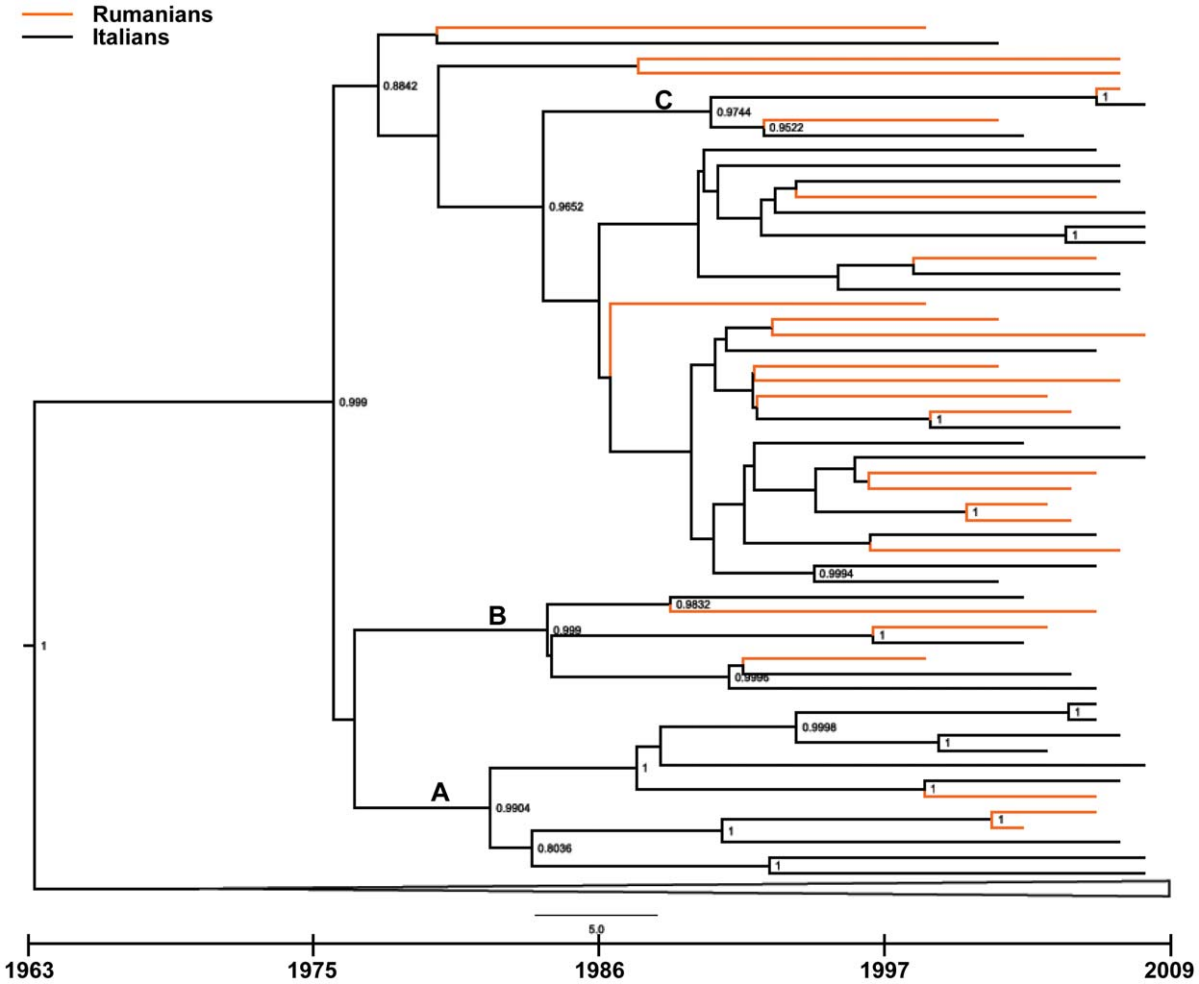
Rumanian cluster details. Time-line scale is displayed underneath the tree. Node labels indicate posterior probability values.

The dated tree is shown in Figure 1. We found a main clade (#2) of 56 patients including all Rumanian patients (n = 25) and 23 of 184 Italian isolates (12.5%). HETs were largely prevalent (91.6%) in this clade; half of patients were females (51.8%) (Table 2). Within this monophyletic group 3 highly significant sub-clades (A, B and C) encompassing 2 or 3 Rumanians and a variable number of Italian isolates (from two to nine each) were identified. The largest cluster including 12 patients dated 27.7 years ago (95% HPD 20.9–68 years ago), the intermediate and the smallest clades contained 7 and 4 isolates and originated 25.4 years ago (95% HPD 20.6–63.7 years ago) and 18.7 years ago (95% HPD 11.8–34.2 years ago), respectively (Figure 2).

**Table 2:** Clusters characteristics and Time of Most Recent Common Ancestor (TMRCA) stratified according to country of origin.

# Clade	Country of origin	Median numberof isolates	% ofRisk Factor	% ofGender	Median TMRCA
2	Italy (n = 23)	56	91.6 HETs	48.1 M	34.2 (22.1–75.4)
	Rumania (n = 25)		8.4 HO-BISEXs	51.9 F	
1,4,9,10,13,15,23,25,27	Italy (n = 34)	5 (3–12)	61.3 HETs	80.8 M	20.5 (17.1–34.7)
	South America (n = 10)		38.7 HO-BISEXs	19.2 F	
3,5,6,7,8,11,12,14,16,17,18,19,20,21,22,24,26	Italy (n = 127)	4 (3–26)	74.6 HETs	81.1 M	18.4 (13.5–36.9)
			25.4 HO-BISEXs	18.9 F	

Nine smaller clades (#1, 4, 9, 10, 13, 15, 23, 25, 27), including a mean of 5 patients (range 3–12 isolates), involved a total of 34 and 10 patients from Italy and South America, respectively. Of note, 8 of 10 South American subjects were from Brazil. Each clade included one patient from the latter geographical area with the exception of clade #25 that encompassed two Italians and two South Americans. HO-BISEXs were 40% of clustering subjects in this group, including 100% of South American patients (Table 2).

The remaining 17 clusters (#3, 5, 6, 7, 8, 11, 12, 14, 16, 17, 18, 19, 20, 21, 22, 24, 26) included only Italian isolates. HETs and HO-BISEXs represented 75.4% and 24.6% of these clustering patients, respectively, of which males were largely prevalent (81.3%).

The mean TMRCA estimation for the tree root was 46.4 years ago (95% HPD 34–106 years ago) implying an origin of the HIV-1 F1 subtype back to the year 1963. Most of the clades originated more than 19 years ago (85.2%), being the cluster with Rumanians the eldest (34.2 years, *p*<.0001). Median TMRCA estimates of different clusters are reported in Table 2. The largest clade containing all Rumanian patients dated 1975 (1934–1987); the median year of origin of the nine clusters involving South American sequences was 1988 (1974–1992) and the Italian clusters originated in 1990 (1972–1996).

### Phylodynamics Analysis

**Figure 3:**
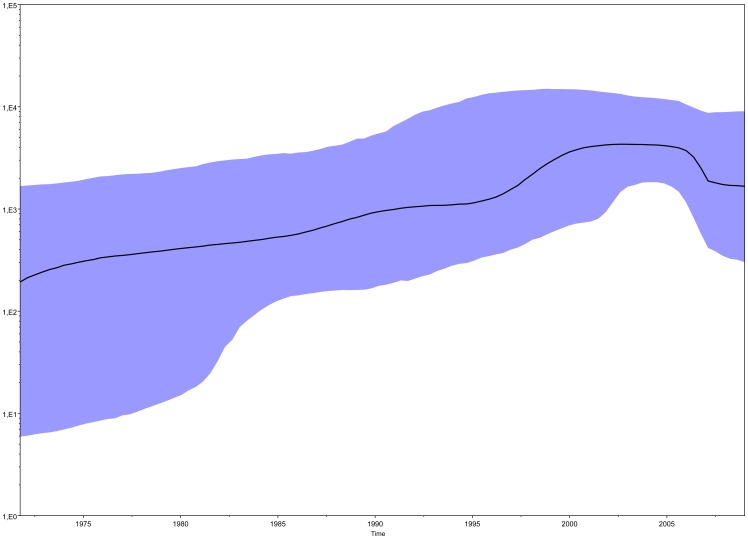
Bayesian Skyline plot of 240 clustering sequences. Ordinate: the number of effective infections at time t (Ne(t)); abscissa: time (in yrs). The thick continuous line represents the median, and the grey area the 95% HPD of the Ne (effective number of infections) estimates.

The evolutionary of population dynamics was estimated in the whole data set. Bayesian Skyline plot is presented in Figure 3 showing the changes in population size at different times from the root of the tree to the time of the most recent isolates (year 2009). The effective number of infections exponentially grew from 1965–1970 to the early 2000s, flattened out until about this year, and then decreased since about 2003.

## Discussion

Although F1 subtype sequences are a relatively low proportion of global clade frequency and, therefore, of the GenBank HIV-1 data, its prevalence may be underestimated due to biased representativeness of geographic areas. Some countries such as Brazil, Argentina and Rumania indeed show high prevalence of this subtype. As a peculiar trait of the non-B Italian epidemic within Europe, F1 clade accounts for more than 40% of non-B circulating isolates [8]. The national ARCA database, currently containing about 3,000 patients with non-B subtypes, allowed us to trace F1 strain epidemic by Bayesian phylogenetic approach with regard to citizenship, mode of infection and gender.

A striking proportion of epidemiological networks (70%) could be identified in our study. A relevant role of sexual networks was highlighted as about 1 of 3 heterosexuals of which 67.7% were man, and about 2 of 3 homosexuals/bisexuals were involved in epidemiological chains. These data extend previous results that demonstrated a high connectivity of contact networks when analyzing transmission events occurring by sexual contacts [18], [19]. By studying a dense population sample of the metropolitan area of Milan we lately found a high proportion of clustering sequences of B subtypes (35%), lower, however, than that detected for F1 isolates in this study. This finding may be related to the characteristics of non-B subtypes circulation in previously B-restricted areas. As a matter of facts, no or a very limited number of new local B variants can be identified at present, while a growing number of local non-B subtypes have been characterized [1]. This may increase the possibility to catch epidemiological networks with non-B clades.

Different modalities of infection in clusters were linked to country of origin. Italian HET males predominated within epidemiological clusters while foreign patients were mainly HET Rumanians, both males and females, and South American HO-BISEXs. The high frequency of Italian HET males and South American HO-BISEXs in epidemiological networks indicated that homosexual or bisexual intercourses have lead to a relevant circulation of F1 subtype in Italy. Despite some reports indicated a partial control of HIV-1 transmission in male to female transsexuals living in Italy, due to condom use [24], marginalized population involved in high risk sexual activity may represent a source of new infections, remaining a specific public health problem.

Phylogenetic analyses are sharp tools to trace potential contact chains, analyze their structure, date their occurrence and correlate them with citizenship risk behaviors and gender. The tree topology indicated that all Rumanian patients segregated in a single clade, including only few Italian individuals, thus suggesting a limited role for Rumanian F1 variant spread among Italians. In contrast, F1 variant from South America had given rise to the Italian F1 epidemic through multiple penetration events, as several independent entry occurrences involved South American and Italian individuals over time.

Some limitations could have affected our work. Firstly, the ARCA database, despite its national coverage and representativeness, like other any other observational database, do not guarantee full records of demographic, clinical and immuno-virological parameters. The relative lack or citizenship and risk factor information for some patients could have weakened the strength of the detected associations. Furthermore, it is well established in the phylogenetic field that the sample density warrants sharp data leading to reliable reporting of clusters. Nevertheless, the frequency of contact networks was exceedingly high in our national cohort despite the probable underestimation related to incomplete data due to intervening individuals not sampled.

As the time-dependent phylogeny is calibrated in calendar years, we were able to estimate when most of the transmissions in each clusters and sub-clusters occurred. Information from dated phylogeny indicated that the entry of F1 clade from Rumania occurred within a long space of time (from 11.8 to 68 years ago) and an internal order of transmission could be defined with high confidence.

A more recent entry could be established for the South American variant, as it occurred likely around 1988 (range: 1974–1992).

We lately established that the spread of F1 subtype among non-B clades was apparently favoured and accompanied with a high frequency of detection of BF genetic mosaic forms in Italy [8]. Both F1 and B/F recombinants may redistribute the relative proportion of the diverse strains possibly leading to overlapping epidemics and conferring to Italy a distinctive picture in the HIV-1 European landscape. Therefore, phylogenetic studies are necessary to elucidate the demographic evolution of other HIV-1 subtypes, more recently introduced in Europe, in order to understand their trend among different ethnicities, risk categories and gender.

Overall, our findings provide new insights to strengthen the surveillance system for HIV-1 and the planning of prevention programs. This study provides sharp information about the association of F1 subtype with geographic origin as well as the sexual routes of transmission among patients living in Italy and carrying such variant. Furthermore, previous data regarding resistance to antiretrovirals in F1 strains circulating in Italy indicate a remarkable frequency of transmitted drug resistance in F1 subtype infected population [25]. Primary resistance in specific clades implies also that, the objective to reduce HIV-1 transmission to low level by an appropriate early usage of antiretrovirals has to take into account such data to develop prevention strategies directed to individuals at high risk behaviors. The study of population dynamics of HIV-1 variants circulating at local level and reconstruction of nationwide transmission networks can contribute to control the epidemic of HIV-1 infection in the next years.

## Supporting Information

Figure S1 Starting tree obtained with MrBayes program showing 27 identified clusters (grey colour).
(TIF)

Table S1 Bayes factor comparison of the parametric and Bayesian skyline plot (BSP) models.
(DOC)
