# Supplementary figures and images for: HIV-1 Subtype F1 Epidemiological Networks among Italian Heterosexual Males Are Associated with Introduction Events from South America
Source: PLoS One. 2012 Aug 2;7(8):e42223. doi: 10.1371/journal.pone.0042223 (PMC3410915; doi:10.1371/journal.pone.0042223)

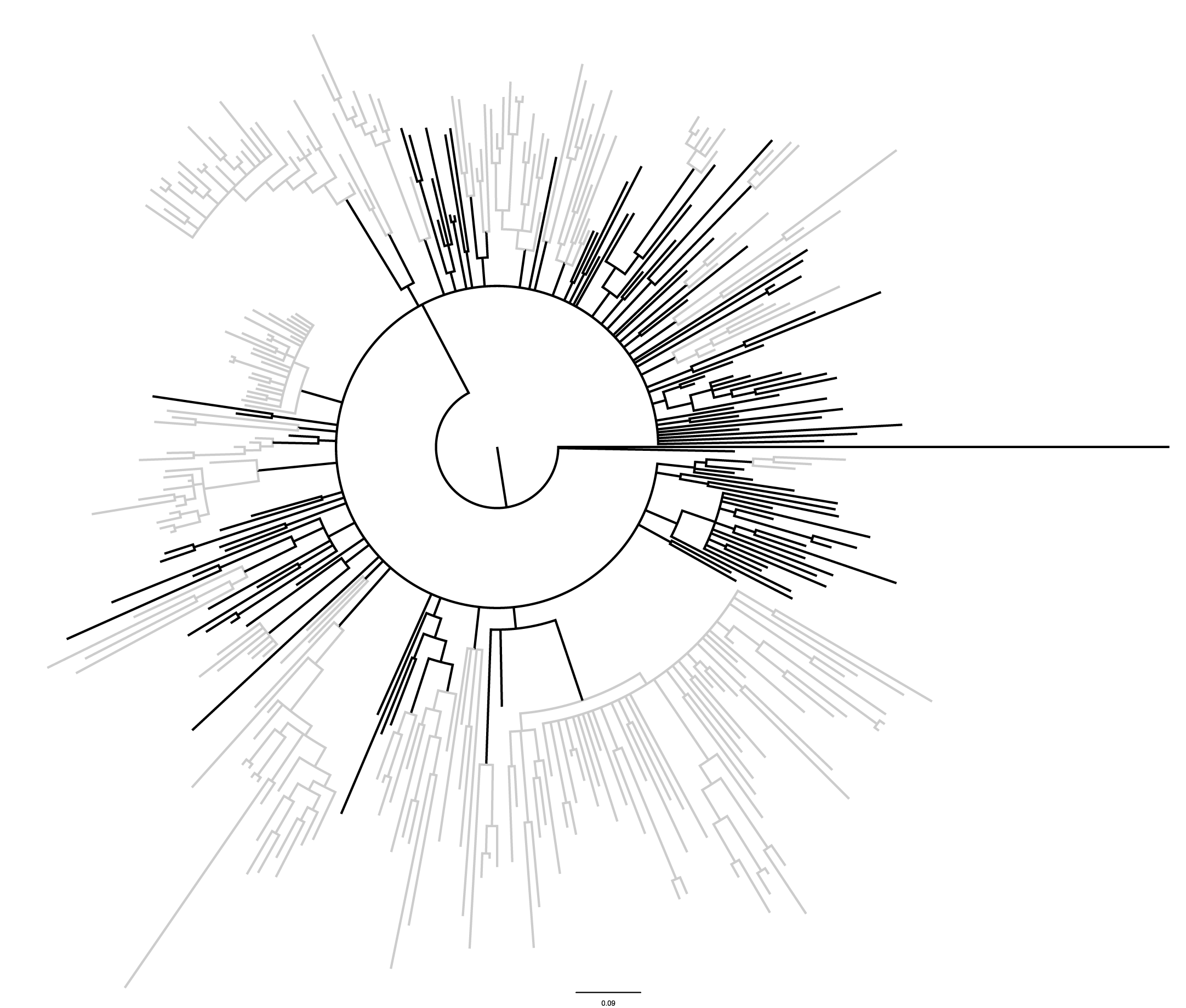

Supplement: Figure S1 — Starting tree obtained with MrBayes program showing 27 identified clusters (grey colour). (TIF) [file pone.0042223.s001.tif]
