# Supplementary material for: HIV-1 Subtype F1 Epidemiological Networks among Italian Heterosexual Males Are Associated with Introduction Events from South America
Source: PLoS One. 2012 Aug 2;7(8):e42223. doi: 10.1371/journal.pone.0042223 (PMC3410915; doi:10.1371/journal.pone.0042223)
Supplement: Table S1 — Bayes factor comparison of the parametric and Bayesian skyline plot (BSP) models. (DOC) [file pone.0042223.s002.doc]

|  | **Constant** | **Exponential** | **Logistic** | **BSP** |
| --- | --- | --- | --- | --- |
| **Constant** | - | -50.06 | -87183.8 | 146.02 |
| **Exponential** | -50.06 | - | -87133.8 | 95.96 |
| **Logistic** | -87183.8 | -87133.8 | - | 87037.8 |
| **BSP** | 146.02 | 95.96 | 87037.8 | - |
